# The effects of default nudges on promoting approval of welfare cuts: An exploration during COVID-19

**DOI:** 10.3389/fpsyg.2022.1038750

**Published:** 2023-01-11

**Authors:** Xin Liu, Ning Zhao, Rui Zheng

**Affiliations:** ^1^Key Laboratory of Behavioral Science, Institute of Psychology, Chinese Academy of Sciences (CAS), Beijing, Beijing Municipality, China; ^2^Department of Psychology, University of Chinese Academy of Sciences, Beijing, China

**Keywords:** policy support, default nudge, opt-in, opt-out, improved opt-out

## Abstract

The global COVID-19 pandemic has created significant financial and operational challenges for some businesses. As a result, temporary welfare benefit reduction may be a tough but future-oriented choice for both employers and employees. The present study examined whether default nudges can be used to promote employees’ approval of welfare-cutting policy while avoiding negative attitudes. Two online surveys were conducted during the first pandemic wave in China (February 2020). In the first study (*N* = 310), the participants were presented with a hypothetical welfare-cutting policy that used either an opt-in approach or an opt-out approach. We aimed to investigate how their approval and attitudes were different between two conditions. The results showed that the employees in the opt-out condition were more likely to accept the welfare-cutting policy than those in the opt-in condition, while participants’ attitudes toward the policy employing opt-out approach were as negative as that employing opt-in approach. Study 2 (*N* = 1,519) involved a replication of Study 1 with two additional improved opt-out approaches (opt-out education and opt-out transparency). Compared with the opt-in approach and standard opt-out approach, the opt-out education approach both increased policy support and improved attitudes toward the welfare-cutting policy. The theoretical and practical implications of these findings are discussed.

## 1. Introduction

Since the emergence of the COVID-19 pandemic at the start of 2020, the virus has caused significant disruptions to the global economy (Nicola et al., 2020). Impacted by economic ripple effects, businesses in various sectors are facing operational challenges and even questions over their survival (Xiang et al., 2021). For example, tourism was unprecedentedly impacted at the onset of the crisis, with airplanes grounded, hotels forced to close, and worldwide travel restrictions (World Tourism Organization, 2020). The sudden stop to tourism has had broad spillover effects on other industries. A report showed that in the 10 months since the initial wave of the pandemic, leisure and hospitality workers experienced a high unemployment rate (Klein and Smith, 2021). Within this collective crisis, an issue of concern might be how to provide a buffer for survival stress and get through the challenge together.

Flexible regulations and good governance are beneficial factors for coping with the crisis. Governments rolled out a series of support policies to ease the economic burden (OECD, 2020; World Trade Organization, 2020). Enterprise can also ease economic pressure in many different ways, such as providing work-from-home options, moving to smaller facilities, reducing pay, and even implementing layoffs (Bartik et al., 2020). Many companies have been forced to rethink employee salary structures (e.g., Ryanair, Kohl’s, and Gap Inc). For example, the HR manager of an organization may adjust the variable performance salary, bonus, and allowance based on the actual working conditions of employees following sufficient negotiation. A poll from the Society for Human Resource Management (SHRM) showed that of 2,200 HR professionals, 19% have decreased pay rates, while another 21% are considering implementing this measure (SHRM, 2020). In China, the Ministry of Human Resources and Social Security (MOHRSS) has advised that if an employer is suffering from production and operational difficulties due to the impact of the pandemic, he or she may consult with employees to adjust their salary (MOHRSS, 2020). While the near-term pain is significant, companies will reduce costs and retain productivity through temporary welfare adjustment, enabling the survival and long-term sustainable development of the enterprise.

In addition to notable economic loss, the COVID-19 outbreak has also caused negative psychological consequences. A series of studies found that people experienced mental health problems during the COVID-19 pandemic, such as anxiety, depression, psychological distress, and even traumatic symptoms (Castelli et al., 2020; Rossi et al., 2020; Ongaro et al., 2021). A meta-analysis revealed that anxiety disorders and depression were prevalent in 33.3%, and 36.3% of the population, respectively, in China (Necho et al., 2021). Employees’ emotion suppression and lack of psychological need fulfillment from the COVID-19 situation were found to impair their work, home, and health outcomes (Trougakos et al., 2020). People and organizations are both under pressure. Organizations should act cautiously against the crisis while minimizing the negative effects on employees’ emotions. Forced cuts in welfare are clearly non-compliant and even invite resentment. It is undoubtedly the case that employees are not very likely to sacrifice their benefits and accept a reduction in welfare payments. How to improve communication with employees is especially significant during a situation of crisis (Sanders et al., 2020; Ayedee et al., 2021). There appears to be a need for an effective and low-cost approach to promote acceptance of the welfare-cutting policy.

Over the past decade, a series of research and policy experience have demonstrated that nudges—choice interventions without forbidding any options or significantly changing their economic incentives—are effective for changing a range of far-sighted behaviors (Thaler and Sunstein, 2008; Benartzi et al., 2017; Venema et al., 2018). Default nudges, as a highly cited example of nudges, have been increasingly used to influence various social issues (Nicolao et al., 2018; Zhao et al., 2022). It can be defined as a switch from an opt-in to an opt-out systems (Blumenthal-Barby and Burroughs, 2012; Reñosa et al., 2021). The opt-in system is also known as an “express consent” policy, requiring individuals to manifestly express their preferences, while the opt-out system assumes that all individuals are willing to accept the preselected option unless they specifically “opt-out” of doing so (Etheredge, 2021). According to the dual process model, nudges may take advantage of the heuristics and biases associated with Type 1 processing (Evans and Curtis-Holmes, 2005; Van Gestel et al., 2020). Changing away from default often involves cognitive effort, and thus people prefer to make choices such that the current state of the world remains intact (Moshinsky and Bar-Hillel, 2010). Default effect could also result from endorsement: people believe that default reflects a trusted recommendation by choice architect (Johnson et al., 2012). Therefore, an option is chosen more often than expected if it is labeled as opting out (Dinner et al., 2011). For instance, in the economic domain, a consumer could be unintentionally manipulated toward accepting the default choice (Brown and Krishna, 2004). Evidence has shown that saving for retirement and insurance consumption increased through setting defaults (Johnson et al., 1993; Madrian and Shea, 2001). In the health domain, defaults often affect policy approval, such as organ donation and transplantation (Johnson and Goldstein, 2003; Abadie and Gay, 2006; Ahmad et al., 2019). Under the opt-out condition, people are more likely to get vaccinated (Liu et al., 2022), accept screening tests for potential diseases (Bartholomew et al., 2020), and choose a healthy diet (Velema et al., 2018). In the organizational context, opt-out approach has also been suggested as an effective intervention to improve employees’ behavior, such as in relation to stand-up working (Venema et al., 2018), pension scheme enrollments (Thaler and Sunstein, 2008; Robertson-Rose, 2021), efficiency in energy use (Brown et al., 2013; Egebark and Ekström, 2016), and flu vaccination (Chapman et al., 2010). Furthermore, default nudges are easy to implement at a low cost (Thaler and Sunstein, 2008), making them suitable to promote policy support.

However, if a default nudge is against people’s will, the strategy could be less effective. For example, Brown et al. (2013) showed that in the case of large decreases in default office thermostat settings, OECD employees did not accept this and restored their preferred thermostat setting. In Venema et al. (2018), employees with stronger intentions to work in a standing position were more likely to approve of the default nudge. Moreover, Colby et al. (2020) demonstrated a dodge effect whereby consumers avoided being nudged if their preferences do not match the default. These findings suggest that people will not be nudgeable when the target behavior conflicts with their preexisting preference. In the current study, supporting a welfare-cutting policy, however, means sacrificing personal interests for the sake of the company’s development. Therefore, employees may already have strong preferences not to participate in welfare-cutting schemes and may not be nudged into the option they do not want. The first exploratory aim of our study was to explore whether changing the default could alter the consent rates for the welfare-cutting policy.

Additionally, there are growing concerns and criticisms about the acceptability of default nudges. As Hausman and Welch (2010) argued, even nudges that maintain nominal freedom of choice may diminish people’s autonomy. Smith et al. (2013) also proposed that even when consequences are benign, default nudge can violate people’s autonomy and their ability to exercise informed choice. A survey reported that default nudges were viewed less favorably and perceived as more threatening to autonomy (Jung and Mellers, 2016). Sunstein (2019) demonstrated that in 12 nudges disapproved of by the majority of Americans, seven involved default rules. Given these critical voices, while changing the default option in the welfare-cutting situation is considered to be a legitimate policy intervention, its acceptability also needs to be evaluated. According to Hagman et al. (2015), nudges can be categorized as pro-self (i.e., focusing on private welfare) or pro-social (i.e., focusing on social welfare) nudges. When compared to pro-self nudges, pro-social nudges were found to be less acceptable and to produce more unfavorable attitudes. Examples of default nudges mentioned above can all be divided into these two categories based on their motivation. Defaults in the welfare-cutting policy, however, cannot be divided into pro-social or pro-self nudges simply. We hypothesized that the policy employing an opt-out approach may not be perceived as acceptable as that employing an opt-in approach in the welfare-cutting scenario.

Improved opt-out approaches have been developed recently in order to address concerns about their acceptability and ethicality perception. For instance, some researchers argued that less transparency might harm the acceptability of defaults (Paunov et al., 2019a,b). This finding suggests that a combination of defaults and transparency information may help change attitudes towards the policy (Bruns et al., 2018; Cheung et al., 2019; Paunov et al., 2020). There is evidence, though, that nudges with transparency do not make people feel more autonomous (Wachner et al., 2020, 2021). Liu et al. (2022) found that a transparent default even decreased people’s perceived freedom to make choices, while opt-out education approach was an effective as well as acceptable intervention to encourage COVID-19 vaccination. Hence, our second aim was to investigate whether improved opt-out approaches (opt-out transparency and opt-out education) could yield a comparable level of effectiveness to the opt-out approach while modifying attitudes towards the welfare-cutting policy.

Employers have been forced to think about cutting welfare in order to save costs and ensure companies’ survivability as a result of the “once-in-a-century” COVID-19 crisis. Such a negative decision may induce resistance and influence employees’ job satisfaction (Kwong and Leung, 2002). We think that default nudge has the potential to provide advice on how to promote acceptance of welfare-cutting policy for emergency management. We attempted to determine an effective way to pitch a welfare-cutting policy to employees and ensure a smooth rollout of the policy. In Study 1, we investigated the effectiveness and perceived acceptability of defaults in the welfare-cutting scenario. Participants were asked to rate whether they agree with the implementation of the given policy and their attitudes towards the policy using opt-in approach or opt-out approach. In Study 2, we aimed to replicate the findings in Study 1 and further explore whether improved opt-out approaches are more acceptable when used to influence employees’ choices.

## 2. Study 1

### 2.1. Materials and methods

#### 2.1.1. Participants

The study was conducted online *via* the popular Chinese professional survey website Wenjuanxing^1^ in February 2020. Participants were recruited by sending the survey link to potential participants *via* the website’s own user recruitment service. To achieve 95% power and to detect a small to medium effect (*ω* = 0.25), a minimum sample size of 208 participants was predetermined using G*Power (Faul et al., 2007). After excluding those who had been unemployed during the survey (*n* = 42), our final sample consisted of 310 employees (mean age = 31.97, *SD* = 7.26). Table 1 provides details of the demographic information. The study was approved by the Institutional Review Board of the Institute of Psychology at the Chinese Academy of Sciences.

**Table 1:** Characteristics of participants in Study 1 (*N* = 310).

Characteristic		*n*	Overall
Gender	Male	152	49.0%
	Female	158	51.0%
Age (years)	18–25	53	17.1%
	26–35	180	58.1%
	> 35	77	24.8%
Education level	High school or below	49	15.8%
	College	233	75.2%
	Master’s or above	28	9.0%
Average monthly income (yuan)	≤ 5,000	74	23.9%
	5,001–10,000	157	50.6%
	> 10,000	79	25.5%

#### 2.1.2. Manipulation and procedure

After providing informed consent, the participants were randomly assigned to the two study conditions (opt-in and opt-out). There was no statistically significant difference between the participants in the opt-in condition and opt-out condition on gender, age, education, or income (all *ps* > 0.50). Following the procedure of intervention described by Yan and Yates (2019), we instructed participants to imagine that their companies or institutions would cut the welfare wages during the pandemic. The following scenario was presented to all participants:

*In the special period marked by fighting against the COVID-19 pandemic, assume that your company/institution plans to implement a new policy of cutting welfare wages in order to alleviate the economic pressure on the company and get through the crisis together. That is to say, the company will pay only the basic salary and cut off welfare wages (that is, the wages paid only include the basic salary, excluding performance wages and other welfare subsidies). The director calls for employees’ opinions on this policy through the company’s website.*

Under the opt-in condition (*n* = 160), participants then read the following statement:

*You will need to submit a request on the website if you agree to the policy implementation. Every employee’s opinion will be respected by the company.*

Under the opt-out condition (*n* = 150), participants read a different statement:

*The company defaults that employees agree to the implementation of this policy, but you are allowed to reject. You will need to submit a rejection request on the company’s website if you disagree with the policy implementation. Every employee’s opinion will be respected by the company.*

Participants then indicated whether they agreed with implementation of the given policy by selecting “yes” or “no.”

Employees’ attitudes towards the policy were also assessed to determine how acceptable the policy they perceived it was. The measure was adapted from Yan and Yates (2019) and Liu et al. (2022), including four items (trust in policymakers, perceived ethicality of the policy, perceived restriction of freedom of choice, and perceived deception and manipulation); a higher score indicated that the given policy is perceived more acceptable. For example, “Please indicate how deceptive and manipulative you think this policy is, ranging from 1 (not at all) to 5 (very much).” Cronbach’s alpha coefficient was used to estimate the internal consistency of the attitude measure (Tavakol and Dennick, 2011). The Cronbach’s α is 0.86 (95% CI [0.83, 0.88]) in Study 1, suggesting that the measure is 86% reliable.

#### 2.1.3. Data analysis

Descriptive analyses were conducted to describe the demographic characteristics. Chi-squared tests were used to test the null hypothesis of perfect randomization in the case of binary variables and independent t test in the case of interval variables.

A chi-squared test was used to preliminarily analyze the hypothesis of the default effect, i.e., there is difference in consent rates between the opt-in approach and opt-out approach. An independent t test was used to compare the mean attitudes between the two conditions. All data were analyzed in R (version 4.1.1; R Core Team, 2022).

### 2.2. Results

Pearson $\chi^{2}$ was used to test the effect of default nudge on percentage of policy supporting behavior (1 = “yes,” 0 = “no”). There was a significant association between condition and whether or not employees agreed with implementation of the policy $\chi^{2}$ (1, *N = 310*) = 8.28, *p* = 0.004. Employees were more likely to agree with the policy that employed opt-out approach (67.33%) than that employed the opt-in approach (51.25%). This appeared to reflect the fact that, according to the odds ratio, there was 1.96 times increase in consent rates for welfare-cutting policy if employees were in the opt-out condition than as opposed to the opt-in condition.

We then performed an independent t-test on the mean scores of the attitude measure. However, there was no statistically significant difference between the opt-in condition (*M* = 2.85, *SE* = 0.08) and the opt-out condition (*M* = 2.98, *SE* = 0.08), *t*(308) = 1.18, *p* = 0.239, *d* = 0.13. Additionally, no significant difference was found on the single item of attitudes between the two conditions, in terms of trust [*t*(308) = 1.41, *p* = 0.159], perceptions of the policy’s ethics [*t*(308) = 0.80, *p* = 0.426], perceptions of how it restricts one’s freedom of choice [*t*(308) = 1.80, *p* = 0.073], and perceptions of deception and manipulation [*t*(308) = 0.02, *p* = 0.982].

### 2.3. Discussion

Study 1 observed the effect of default nudge on policy-supporting behavior. However, the opt-out approach appeared to be as poorly acceptable as the opt-in approach (on the five-point scale, employees’ mean attitude scores were close to the midpoint of the scale for both approaches). Previous studies found that compared with those in the opt-in condition, people who were presented with the opt-out approach expected to be less satisfied with their choice (Wachner et al., 2021) and perceived higher deceptiveness (Paunov et al., 2019a), results that were inconsistent with those in the present study. On the one hand, the background and scenario in the current study were different from the existing research. Uncertainty plagues people during times such as the ongoing COVID-19 pandemic (Sharma et al., 2020; Merlo et al., 2021). Welfare-cutting policy employed with opt-out approach may not be maybe less acceptable than that employed with opt-in approach. On the other hand, insufficient data may be one factor in the failure to identify a statistically significant mean difference in attitudes. The priori sample size in Study 1 was only determined based on chi-squared test so that there may not be enough statistical power to detect a true effect in t-test analysis. Therefore, in Study 2, we recruited a larger sample and further examined whether there was an attitude difference between the opt-in approach and opt-out approach and whether the improved opt-out approach could increase perceived acceptability of the welfare-cutting policy.

## 3. Study 2

### 3.1. Materials and methods

#### 3.1.1. Participants

The participant recruitment and inclusion criteria were similar to those in Study 1. Given that the actual effect size on attitudes difference was only *d* = 0.13 and the actual effect size on supporting rate difference achieved at least 90% statistical power, the parameters of the sample size calculation in Study 2 were changed. Study 2 aimed to detect a relatively small effect size (*d* = 0.20 and *f* = 0.10) at 90% chance. G*Power analysis showed that at least 356 participants would be required for each condition, i.e., the recommended total sample size was *N* = 1,424. A total of 1787 participants completed the survey, with 1,519 valid responses. Their average age was 31.74 years (*SD* = 7.24), range = 18 years ~69 years. Details of the demographic information are provided in Table 2. The study was approved by the Institutional Review Board of the Institute of Psychology at the Chinese Academy of Sciences. Informed consent was provided by all participants.

**Table 2:** Characteristics of participants in Study 2 (*N* = 1,519).

Characteristic		*n*	Overall
Gender	Male	618	40.7%
	Female	901	59.3%
Age (years)	18–25	262	17.2%
	26–35	897	59.1%
	> 35	360	23.7%
Education level	High school or below	326	21.5%
	College	1035	68.1%
	Master’s or above	158	10.4%
Average monthly income (yuan)	≤ 5,000	360	23.7%
	5,001–10,000	766	50.4%
	> 10,000	393	25.9%

#### 3.1.2. Manipulation and procedure

The participants were randomly assigned to one of the four policy conditions, including an opt-in condition, a standard opt-out condition, and two improved opt-out versions (opt-out education and opt-out transparency). There was no statistically significant difference among the four conditions on gender, age, education, or income (all *ps* > 0.35).

Instructions were similar to those of Study 1. The opt-in condition depicts a policy form in which people are assumed to not be willing to participate in the welfare-cutting plan unless they actively register, whereas the opt-out conditions present a policy form that assumes enrollment unless people choose to deregister. In addition, the opt-out transparency condition explained the purpose and the fact that people may be subconsciously affected by the default in the opt-out condition (i.e., “*This policy defaults that all employees are consenting to the welfare-cutting plan, which may influence your choice. The purpose of the policy is to increase employees’ approval for the policy, thereby alleviating the company’s financial stress during the pandemic.*”). The opt-out education condition highlighted that educational information will be provided to promote understanding about the policy (i.e., “*Note. Welfare cutting is an important temporary measure to decrease costs and increase efficiency during the period against the epidemic, and the original welfare policy will be restored after the resumption of work. The company hope to get through the current dilemma together with you.*”). More details of the four conditions are presented in Table 3.

**Table 3:** Differences across opt-in condition, opt-out condition, and opt-out improvements.

Condition	Differences and improvements
Opt-in	If no choice is registered, then employees are assumed to be unwilling to participate in the designated welfare-cutting plan.
Opt-out	If no choice is registered, then employees are assumed to be willing to participate in the designated welfare-cutting plan.
Opt-out transparency	Based on the opt-out approach, the company also explains the goal and behavioral consequences of this opt-out approach to its employees.
Opt-out education	Based on the opt-out approach, the company also advertises the need to cut welfare and educates employees about the designated welfare-cutting plan.

After making their choice on whether they agreed or disagreed with the welfare-cutting policy implementation, the participants completed similar attitude measures as in Study 1: (a) trust in policy-makers, (b) perceived ethicality of the policy, (c) perceived restriction of freedom of choice, and (d) perceived deception and manipulation. Cronbach’s *α* is 0.84.

#### 3.1.3. Data analyses

The descriptive analyses and randomization checks were similar to those in Study 1. Logistic regression was conducted to identify the influence of the approaches that policy on acceptance. Then, we performed a one-way ANOVA to test whether attitudes changed among conditions.

### 3.2. Results

#### 3.2.1. Effectiveness of the default nudges

**Figure 1:**
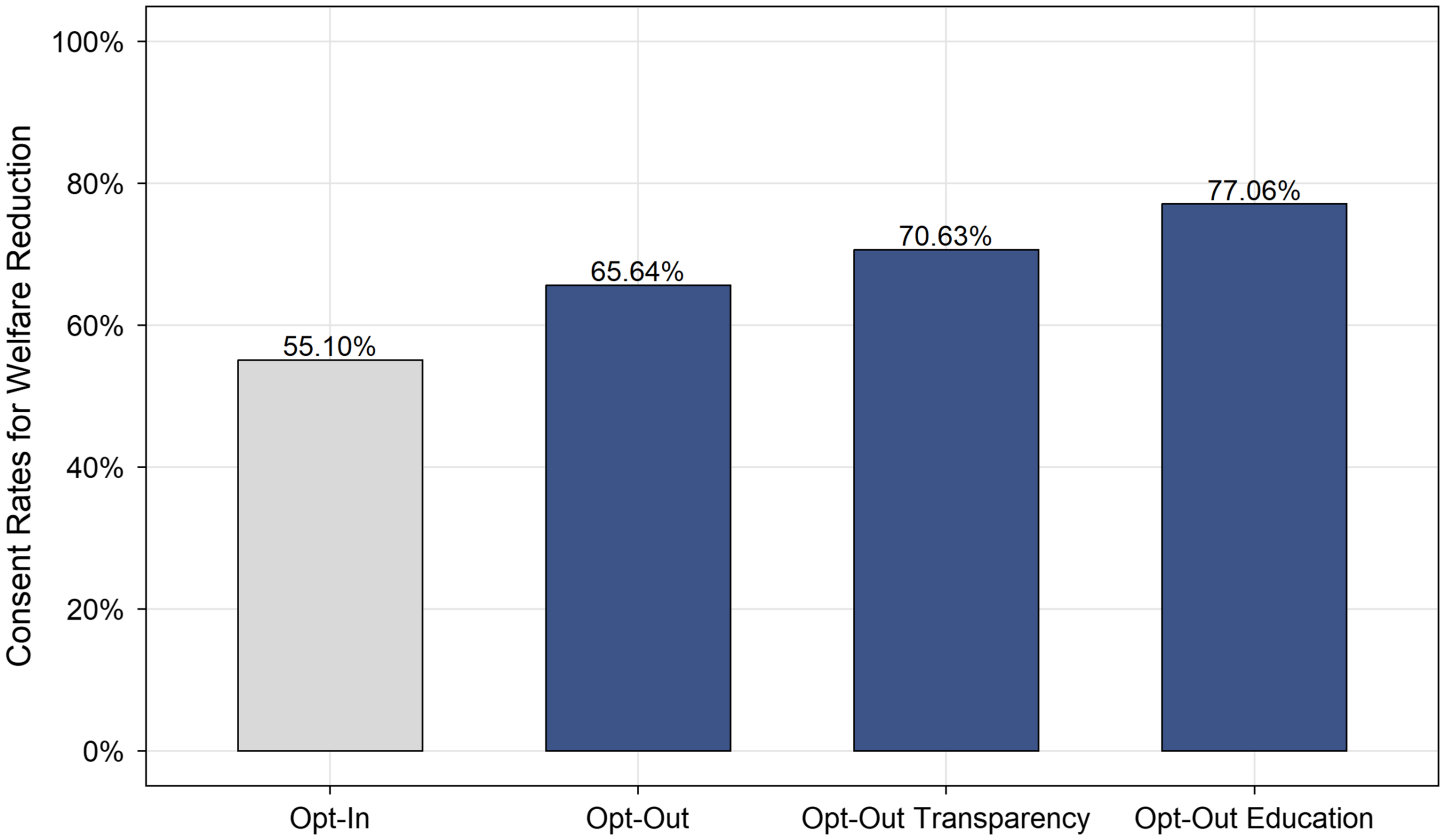
Consent rates for welfare reduction by conditions of policy (Study 2).

Figure 1 presents the acceptance of the welfare-cutting policy among the four conditions. Employees were more likely to agree with the policy using the opt-out approach (65.64%) and the policy using its improved approaches (70.63% and 77.06%) than with the opt-in welfare-cutting policy (55.10%). Making “agree” the default option increased the consent rate for the welfare-cutting policy by at least 10%. The logistic regression test showed that the approaches used by the welfare-cutting policy significantly affected the consent rates. As shown in Table 4, compared with the opt-in approach, the opt-out approach increased participants’ level of consent for the welfare-cutting policy (OR = 1.56). Moreover, people in the condition using the opt-out transparency approach (OR = 1.96) and in the condition using the opt-out education approach (OR = 2.74) are more likely to agree with the implementation of the policy.

**Table 4:** Results of the binary logistic regression analysis.

	Estimate	OR	SE	*p* Value	95% CI
Opt-in	Ref				
Opt-out	0.44	1.56	0.15	0.003	0.15–0.74
Opt-out transparency	0.67	1.96	0.15	< 0.001	0.37–0.98
Opt-out education	1.01	2.74	0.16	< 0.001	0.70–1.32

*R*^2^ = 0.02 (Hosmer-Lemeshow), 0.03 (Cox-Snell), 0.04 (Nagelkerke), Model *χ*^2^(3) = 43.78, *p* < 0.001.

To compare the opt-out approach and its improved versions, we also established the standard opt-out approach as the reference condition. The model with the opt-out approach as a baseline also was better than chance at predicting the outcome, $\chi^{2}$ (3) = 43.67, *p* < 0.001. The results showed that the opt-out education approach significantly increased consent rates compared to the standard opt-out condition (OR = 1.76, *β* = 0.56, *p* < 0.001, 95% CI [0.25, 0.88]). However, there was no significant difference in consent rates between the opt-out transparency condition and the opt-out condition (*β* = 0.23, *p* = 0.131, 95% CI [−0.08,0.53]).

#### 3.2.2. Attitudes towards default nudges

ANOVA showed that approaches employed by welfare-cutting policy affected participants’ attitudes, *F*(3, 1515) = 4.72, *p* = 0.003, $\eta_{partial}^{2}$ = 0.01. As in Study 1, no statistically significant difference of attitudes was detected between opt-in approach and opt-out approach, *β* = 0.005, *SE* = 0.07, *p* = 1.000, 95% CI [−0.16, 0.17]. However, employees perceived the opt-out education approach as more acceptable than the standard opt-out approach (*β* = 0.19, *SE* = 0.06, *p* = 0.017, 95% CI [0.02, 0.35]) and opt-in approach (*β* = 0.18, *SE* = 0.07, *p* = 0.024, 95% CI [0.02, 0.35]). We also analyzed the differences in the single-item attitude measure. Compared with the standard opt-out approach, the policy implementing opt-out education approach also increased perceived trust towards the company (*β* = 0.26, *SE* = 0.08, *p* = 0.003, 95% CI [0.07, 0.46]) and perceived ethicality (*β* = 0.27, *SE* = 0.08, *p* = 0.005, 95% CI [0.06, 0.47]). However, there was no statistically detectable effect of approaches on employee’s perceptions of how the policy restrict freedom of choice [*F*(3, 1,515) = 1.40, *p* = 0.246] and perceptions of deception and manipulation [*F*(3, 1,515) = 1.30, *p* = 0.274]. Details of the condition means are provided in Table 5.

**Table 5:** Condition means and standard errors of acceptability measure.

	Opt-in	Opt-out	Opt-out transparency	Opt-out education
	(*n* = 363)	(*n* = 390)	(*n* = 378)	(*n* = 388)
Composite measure	2.91(0.05)	2.91(0.05)	3.10(0.05)	3.06(0.05)
	Single item				
Perceived trust	3.04(0.06)	3.07(0.06)	3.29(0.05)	3.33(0.05)
Perceived ethicality	3.01(0.06)	2.95(0.06)	3.17(0.06)	3.21(0.06)
Perceived freedom of choice	2.74(0.06)	2.70(0.06)	2.83(0.06)	2.83(0.05)
Perceived deception and manipulation	2.86(0.06)	2.91(0.05)	2.96(0.06)	3.01(0.06)

### 3.3. Discussion

In Study 2, we replicated the default effect in the welfare-cutting scenario, i.e., opt-out approaches (especially opt-out education approach) increased approval of the policy. This study also aimed to test whether improved opt-out approach can increase policy acceptance. The results indicated that participants considered the opt-out education approach as more acceptable, especially regarding trust in policymakers and perceived ethicality of the policy.

## 4. General discussion

Businesses are facing difficult decisions under the threat of COVID-19, and many may need to reduce workers’ welfare or pay to stay afloat. Low-cost and effective solutions are needed to promote consent for the welfare-cutting scheme in such an emergency. The purpose of the current two studies was to investigate the effects of default nudges in the welfare-cutting scenario. This was done with the opt-out approach that defaults participants’ consent to the welfare scheme and the opt-in approach that was without any pre-selected option. Additionally, we tested whether employees’ perceptions of the policy was influenced by improved opt-out approaches.

First, both Study 1 and Study 2 showed that participants who were presented with an opt-out approach were more likely to agree with the implementation of the welfare-cutting scheme. In the COVID-19 pandemic context, default-based intervention has been increasingly used to nudge people into the desired behavior and increase policy adherence. For example, nudging *via* prescheduled appointments or switching the default option increased COVID-19 vaccination rates (Bonander et al., 2022; Tentori et al., 2022). A narrative review indicated that nudging strategies, including default rules, could be important tools for improving the treatment of COVID-19 (Vilhelmsson et al., 2021). However, previous attempts focused on improving health decision-making relevant to COVID-19. To our knowledge, this study is the first to explore how default nudges can be used to affect policy adherence in the workplace during the pandemic. The effectiveness of the default nudge on support for the welfare-cutting policy is consistent with its known successes in the workplace, for example, regarding stand-up working (Venema et al., 2018), saving behavior (Madrian and Shea, 2001), electricity consumption (Brown et al., 2013), and productivity of knowledge workers (Ebert and Freibichler, 2017). As Dianoux et al. (2019) proposed, nudges facilitate agile communication in a changing situation. Our findings support the notion that default nudges can be leveraged by HR managers to encourage policy approval in the face of turbulence and uncertainty.

Why can welfare-cutting policy adherence (at least on intention) be nudged by a default option? There are some potential explanations. For example, in the framework proposed by Löfgren and Nordblom (2020), choices are made in two steps. In Step 1, individuals decide whether the choice should be made attentively or inattentively. If one makes an inattentive choice, one’s actual choice may be susceptible to nudges in Step 2. Nudgeability seems to coincide with the degree of unimportance of the choice because the more important the choice, the more attention it should be given. Putting the default effect we found under this framework, however, the decision on whether to support the welfare-cutting policy cannot be unimportant. As the authors indicated, an important choice made by a low-confidence decision-maker also can be made inattentively. In the welfare-cutting situation, the effort required to make an attentive choice may be very high, and employees may have a low degree of confidence that their choice will be the preferred one. Therefore, employees’ approval of welfare-cutting policy can be nudgeable. Additionally, de Ridder et al. (2022) indicated that nudge effects do not hinge on modes of thinking but that personal preferences moderate nudging effects. They proposed an inverted U curve to capture the relation between preexisting preferences and nudgeability. Under their hypothesis, employees with a medium level of preference for welfare-cutting policy have been nudged by the default option. Together, these explanations call for greater scrutiny of the theoretical model of nudges. Future research needs to explore the underlying mechanisms that drive individuals’ susceptibility to nudges.

Second, the implementation of a welfare-cutting policy aids businesses in navigating uncertainty but should take into account the attitude of employees. We detected no evidence that opt-out approach decreasing employees’ attitudes. Rather, it yielded similar attitudes to those of the opt-in approach. Likewise, there is evidence that shows that default nudges may not be as manipulative and autonomy threatening as we feared (Michaelsen et al., 2021; Liu et al., 2022). Previous studies have shown that pro-self nudges are more popular than pro-social nudges (Hagman et al., 2015, 2022), while this result could vary depending on who does the nudging (Tannenbaum et al., 2017) and who is nudged (Jung and Mellers, 2016). For example, Arad and Rubinstein (2018) found that the attitude toward nudge was somewhat more positive when it was implemented by one’s employer rather than by the government. That is, there are relative preferences for employers’ nudges. Although we did not compare attitudes towards nudges when they are implemented by the government with those when they are implemented by an employer, our findings suggested that default nudges implemented by employers on welfare cuts during COVID-19 did not appear to harm attitudes. Opting-out of welfare reduction does not seem to be a pro-self nudge, but it is favorable to the whole company during the pandemic. Employees, as stakeholders of the collective, may find the policy acceptable because it will be beneficial to their future development.

Furthermore, Study 2 revealed that the improved opt-out approaches did not undermine the policy’s effectiveness, and employees in the opt-out education approach were even more likely to approve of the policy than the opt-out approach. In terms of attitudes, the opt-out approach with education received the least objections, especially regarding trust in policymakers and perceived ethicality. This finding suggests that people’s concerns about the welfare-cutting policy likely come from a lack of understanding, and thus educating people about why the policy is being proposed may promote employees to comprehend and accept the opt-out approach. Liu et al. (2022) also demonstrated that a vaccination opt-out education approach was the most acceptable among the five approaches presented. However, in Yan and Yates (2019), people’s perceived autonomy and freedom of choice for opt-out education were less than those for the opt-in approach in the carbon emission offset policy domain, while opt-out education yielded a comparable level of acceptability to the opt-in approach in the retirement saving domain. In addition, improvement in education made people conscious that retirement saving was a social issue, but was found to be less effective in resolving negative emotions and uneasiness due to the company’s intervention (Yan and Yates, 2019). These findings suggest that education may lead to attitude change, but it can also trigger resistance. When applying the opt-out education to real-world scenarios, policymakers need to carefully examine the particular policy to decide whether our results have practical application.

Naturally, there are some limitations of the study. First, China has been labeled as a “nudge enthusiast,” in which overwhelming majorities approve of nearly all nudges (Sunstein et al., 2019). Jung and Mellers (2016) found that those who were more individualistic were more inclined to disapprove of companies that implemented nudges. It will be interesting to explore the effectiveness and employees’ perceptions of the welfare-cutting opt-out approach under an individualistic culture. Second, although we emphasize the context of COVID-19 crisis and infer that the effect of default nudges during the pandemic may not be generalizable to other normal circumstances, it will be interesting to examine whether default nudges are still effective without a pandemic background. For example, if a company plans to cut welfare due to poor management, will employees be more likely to accept the policy employing an opt-out approach? In addition, our findings on the welfare-cutting policy domain may not be generalizable to other policy domains. Policy-makers are supposed to avoid using a default nudge excessively or inappropriately. Third, we examined employees’ approval of policy instead of their actual behavior. While there is evidence suggesting that default policies affect hypothetical and actual behavior in a similar way (Momsen and Stoerk, 2014; Venema et al., 2018), there also can be a gap between behavioral intention and actual actions. Campos-Mercade et al. (2021) found that nudges of social impact and argument increased participants’ intentions to get COVID-19 vaccinations, but none increased actual vaccination uptake. Lee et al. (2018) invoked social norms to nudge HPV vaccinations. In their study, daughters in the intervention group reported a higher intention to vaccinate, while vaccine uptake was the same between the nudge-intervention group and the control groups. Employees lose nothing when approving a hypothetical welfare-cutting scheme, which implies that observation of the effect of default nudges on promoting intentions may not generalize to the real world. The potential intention–behavior gap calls for more studies to investigate whether there are default effects on actual enrollments in the welfare-cutting scheme.

The recommendations for the future research proposed in this study are as follows. First, there may be other factors that could influence or explain employees’ final choice on welfare-cutting policy, such as income and job experience. Future research could investigate whether these factors play a moderation role in default effects. Second, the opt-out education approach improved attitudes and acceptance of the policy. It is worthwhile to explore how people perceived opt-in and opt-out approaches in order to understand why enhancement of the opt-out approach works. Finally, another possible direction for future research is related to gathering evidence of nudge interventions at workplace. Here, we provided some preliminary insight regarding the roles of default nudges in advancing welfare-cutting policy during the pandemic. Future research could be extended to the normalized situation and other nudging approaches.

Under the collective crisis of COVID-19, policy interventions based on behavioral economics insights are simple and cost-effective ways to promote employees’ policy support. This paper is an attempt to explore how default nudges can be leveraged to promote approval of a welfare-cutting policy during the crisis. Our findings provide evidence that the use of a default nudge increases employees’ approval of the policy, and an opt-out approach that emphasizes education could enhance their trust in employers and their perceptions of policy’s ethicality. Therefore, we recommend including education describing the policy to employees in order to mitigate their objections to the policy.

## Data availability statement

The raw data supporting the conclusions of this article will be made available by the authors, without undue reservation.

## Ethics statement

The studies involving human participants were reviewed and approved by Institutional Review Board of the Institute of Psychology at the Chinese Academy of Sciences. Written informed consent for participation was not required for this study in accordance with the national legislation and the institutional requirements.

## Author contributions

NZ and RZ contributed to the conception and design of the study. NZ organized the database. XL performed the statistical analysis and wrote the first draft of the manuscript. XL and RZ contributed to the manuscript revision, read, and approved the submitted version.

## Funding

This work was funded by the National Natural Science Foundation of China (Grant No. 71771209) and the Major Program of the National Social Science Foundation of China (Grant No. 19ZDA358).

## Conflict of interest

The authors declare that the research was conducted in the absence of any commercial or financial relationships that could be construed as a potential conflict of interest.

## Publisher’s note

All claims expressed in this article are solely those of the authors and do not necessarily represent those of their affiliated organizations, or those of the publisher, the editors and the reviewers. Any product that may be evaluated in this article, or claim that may be made by its manufacturer, is not guaranteed or endorsed by the publisher.

## Footnotes

^1^ www.sojump.com
